# Epidermal Growth-Factor – Induced Transcript Isoform Variation Drives Mammary Cell Migration

**DOI:** 10.1371/journal.pone.0080566

**Published:** 2013-12-06

**Authors:** Wolfgang J. Köstler, Amit Zeisel, Cindy Körner, Jonathan M. Tsai, Jasmine Jacob-Hirsch, Nir Ben-Chetrit, Kirti Sharma, Hadas Cohen-Dvashi, Assif Yitzhaky, Eric Lader, Ulrich Tschulena, Gideon Rechavi, Eytan Domany, Stefan Wiemann, Yosef Yarden

**Affiliations:** 1 Departments of Biological Regulation, Weizmann Institute of Science, Rehovot, Israel; 2 Physics of Complex Systems, Weizmann Institute of Science, Rehovot, Israel; 3 Division of Molecular Genome Analysis, German Cancer Research Centre (DKFZ), Heidelberg, Germany; 4 Sheba Cancer Research Center, The Chaim Sheba Medical Center and Sackler School of Medicine, Tel Aviv University, Tel Aviv, Israel; 5 Qiagen, Frederick, Maryland, United States of America; Rikagaku Kenkyūsho Center for Allergy and Immunology, Japan

## Abstract

Signal-induced transcript isoform variation (TIV) includes alternative promoter usage as well as alternative splicing and alternative polyadenylation of mRNA. To assess the phenotypic relevance of signal-induced TIV, we employed exon arrays and breast epithelial cells, which migrate in response to the epidermal growth factor (EGF). We show that EGF rapidly – within one hour – induces widespread TIV in a significant fraction of the transcriptome. Importantly, TIV characterizes many genes that display no differential expression upon stimulus. In addition, similar EGF-dependent changes are shared by a panel of mammary cell lines. A functional screen, which utilized isoform-specific siRNA oligonucleotides, indicated that several isoforms play essential, non-redundant roles in EGF-induced mammary cell migration. Taken together, our findings highlight the importance of TIV in the rapid evolvement of a phenotypic response to extracellular signals.

## Introduction

Analyses of the human transcriptome revealed that most genes are transcribed into several distinct mRNA isoforms [1], [2], [3], [4]. Transcript diversity is generated by interrelated epigenetic, co-transcriptional and post-transcriptional mechanisms, including alternative promoter usage, changes in the editing, methylation, splicing and polyadenylation of mRNA [5], [6], [7]. These processes regulate mRNA and protein abundance by affecting sequences recognized by RNA-binding proteins or non-coding RNAs, as well as by means of modifying translation efficacy. Altogether, these processes are thought to immensely increase the diversity of transcriptomes and proteomes. Accordingly, transcript isoforms derived from the same gene may exhibit distinct, sometimes even opposing functions [8], [9]. Signals induced by cell adhesion, stimulation of nuclear and immune receptors, as well as oncogenes and tumor suppressor genes, all have been shown to regulate the cellular machineries governing mRNA diversity [10], [11], [12], [13], [14], [15], [16]. The resulting transcript isoform variation (TIV) is mediated by activation of canonical signaling pathways, such as the phosphatidylinositol 3-kinase – AKT pathway. Prototypical TIV-inducing stimuli include growth factors, such as hormones and the epidermal growth factor (EGF). For instance, EGF-activated AKT signals stimulate a protein kinase specific for the family of serine/arginine-rich (SR) regulators of mRNA splicing [12].

Previous transcriptome-wide studies analyzing stimulus-induced TIV focused predominantly on immune cells [17], [18], [19], [20]. Likewise, hypoxic stress and androgen stimulation were shown to generate, after 24 hours, widespread TIV in endothelial and prostate cancer cells, respectively [21], [22]. Shorter stimuli, such as thrombin (6h) or insulin (5h), have also been reported to induce TIV in pulmonary endothelial cells and in *Drosophila* S2 cells, respectively [23], [24]. Furthermore, analysis of chromatin immunoprecipitates using antibodies to RNA polymerases and promoter tiling arrays demonstrated widespread alternative promoter usage in a breast cancer cell line, three hours after treatment with estradiol [25]. A single study used a time course experiment, rather than one or two post-stimulus time points, to profile depolarization-induced TIV in neuroblastoma cells [26].

In aggregate, available information on the dynamics and other features of inducible TIV events is scarce, and their functional relevance remains incompletely understood. For instance, stimuli might induce a simple permanent switch of transcript isoforms similar to the TIV events induced by developmental cues, which regulate lineage commitment [8], [27]. Conversely, transient stimulus-induced TIV events might represent either transcriptional noise or, as previously shown for gene-expression changes following stimulation [28], represent an essential part of an ordered cascade of transcriptional events. The EGF receptor (EGFR) represents one of the best characterized regulators of transcription and fate decisions taken by epithelial cells. Accordingly, perturbations impinging on EGFR are causally implicated in many diseases, particularly cancer [29]. Therefore, the present study assumed that EGFR signaling can provide an important framework for identifying signal-induced TIV and for understanding its functional ramifications.

## Results

### EGF rapidly induces widespread non-monotonous TIV

**Figure 1:**
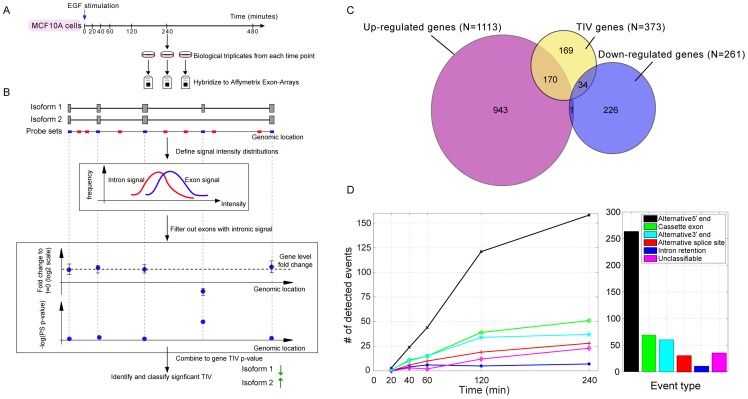
EGF induces time-dependent TIV in mammary cells. (**A**) *Experimental design:* Biological triplicates of MCF10A cells were stimulated with EGF (20 ng/ml) for the indicated intervals and each sample was hybridized separately to Affymetrix Human Exon 1.0 arrays. (**B**) Analysis outlines. *Upper panel:* For each gene, both the annotation and probe set (PS) information were used to define the PS as interrogating either constitutive introns (red) or putative exons (blue). *Middle panel:* Only putative exonic PS whose signal intensities were significantly higher than the introns' were considered. *Lower panel:* At each time point, differentially expressed genes were identified as follows: Fold change (FC) of the signal intensity between unstimulated and stimulated cells was calculated for all exonic PS. Gene-level FC (dashed line) is the median exonic FC. EGF-induced TIV events were identified whenever FC of exonic PS behaved differently from the gene-level FC; statistical significance was assessed using FDR analysis. (**C**) Venn diagrams showing the number of significantly (FDR<5%) differentially expressed genes, with FC ≥1.5, along with the number of genes for which TIV took place (FDR<5%) at two or more adjacent time points. One gene, *WEE1*, was found to be significantly up-regulated at early time points following EGF stimulation and down-regulated towards the end of the time course. (**D**) Histograms showing the number of TIV events present at different time points and the cumulative number of events following an EGF stimulus, separately for different types of TIV events. For all analyses, only TIV events detectable in at least two adjacent time points were considered. Hence, TIV events detectable 480 min after stimulation are not displayed because they had to be present at 240 minutes as well.

To characterize signal-induced TIV, we used MCF10A mammary cells, which migrate in response to EGF stimulus [30], [31], [32]. Starved MCF10A cells were stimulated with EGF, total RNA was isolated from biological triplicates at seven time points, and samples were individually hybridized to exon arrays (Figure 1A). These microarrays encompass 1.4 million probe sets (PS), which interrogate the expression of known and putative exons. Notably, PS interrogating intronic transcript regions closely reflect pre-mRNA expression, while exonic signals represent the more abundant mature mRNAs [33]. To exclude spurious signals from introns in a biological system that only initially is at transcriptional steady state, we developed an algorithm that identifies truly exonic transcript regions under such conditions (Figure 1B, Figure S1, and Information S1). Next, exons that concordantly varied over time, and were also shared by the prevalent isoforms, were used to define gene-level fold changes (FC). Conversely, exons that behaved in a non-concordant way in at least two adjacent time points were used to identify TIV events.

Our analyses revealed that EGF induced up- or down-regulation of a substantial fraction of the genes expressed by MCF10A cells (1373 out of 7968 genes, 17%). These analyses employed a 1.5-fold change (FC) cut-off on top of a false discovery rate (FDR) threshold <5% to detect only those significantly differentially expressed genes, which exhibited a sizeable FC (see *Materials and Methods*). Applying the same numerical FDR threshold (i.e., 5%), a large number of genes were identified to exhibit TIV (n = 373; see Information S1), many of which were not differentially expressed upon stimulus (Figure 1C). The numbers of genes identified as differentially expressed or undergoing TIV in our experiments were similar to those reported in other studies investigating stimulus-induced TIV [19], [21], [22], [26]. As shown in the Information S1, functional enrichment analysis did not identify clearly enriched biological processes, specific molecular functions, cellular components or pathways in the set of genes undergoing TIV. Our time-course experiment allowed us to classify, for each time point, the abundance of respective types of TIV events and their peak times. These analyses revealed that EGF-induced TIV occurs rapidly and exhibits non-monotonous behavior, with a considerable number of TIV events present within the first hour after stimulus, but the majority of events emerging toward the end of the stimulation period (Figure 1D, File S1 sheet 1). Notably, in similarity to differentially expressed genes, some TIV events exhibited a transient behavior, whereas other TIV events persisted throughout the examined time course. Surprisingly, although likely driven by different yet interrelated molecular ensembles of the cellular machineries involved in transcription (e.g., alternative promoter usage) and RNA splicing (e.g., cassette exon events), the peak times of different TIV event types exhibited remarkably similar temporal distributions. The most frequent event type we observed was alternative 5′ transcript ends, resulting from either alternative first exon usage or alternative transcription start sites within the same first exon.

### EGF-induced promoter switching

**Figure 2:**
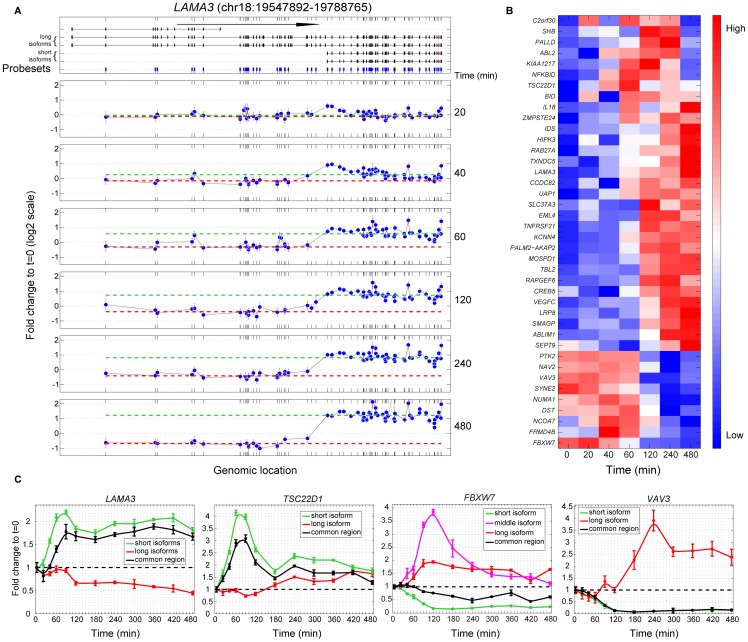
EGF-induced alternative promoter usage. (**A**) *Upper panel:* Organization of *LAMA3* transcripts as defined by the UCSC gene model. Vertical boxes represent exons; the arrow (100 kb) indicates the direction of transcription. Positions of exonic PS (blue) that passed all filtering steps are indicated. The upmost isoform was not expressed in MCF10A cells (neither of two isoform-specific PS interrogating the alternative last exon passed the required signal intensity threshold). *Lower panels:* FC (log2 scale) of each PS with respect to pre-stimulus values is shown for each time point. Only PS with ‘present’ calls in all replicates are depicted at each time point. Bars represent standard errors of FC from three biological replicates. Note strong induction of the 3′ portion (the dashed green line marks the median FC of this region). In contrast, the longer isoforms (median FC shown by the dashed red line) are slightly downregulated. (**B**) The heat map displays the FC ratios of the short to long isoforms of the top 40 genes identified as undergoing EGF-induced alternative usage of well-annotated promoters (FDR<1%) resulting in alternative first exons; if the relative abundance of the short isoforms compared to the long isoforms increases after EGF stimulation the FC ratio becomes ‘high’ (see for instance *LAMA3*) (**C**) For qPCR validation of TIV events primer pairs specific to transcript isoforms were used for each gene, along with pairs spanning regions common to all transcripts. Error bars represent standard deviations from three technical replicates. The experiment was repeated twice.

We first focused on EGF-induced alternative promoter usage. Intron profiles of the corresponding isoforms indicated regulation at the level of transcription, rather than by differential stabilization of transcripts with distinct 5′ ends (data not shown). The temporal profile of a typical TIV event emanating from switching between well-annotated alternative promoters is shown in Figure 2A for the laminin alpha 3 (*LAMA3*) gene, encoding a basement membrane component; the long *LAMA3* isoforms (herein termed isoform 1) were downregulated, whereas the short isoforms (herein: isoform 2) were significantly induced. Likewise, Figure 2B presents temporal profiles of the ratios between short and long isoforms of 40 different genes. From the list of 373 TIV events, the latter represent the top ranked (FDR<1%) EGF-induced alternative promoter usage events resulting (a) from switching between well-annotated (see http://genome.ucsc.edu) alternative promoters, and (b) in isoforms with distinct first exons (rather than different transcription start sites within the same first exon only).

We next validated several microarray-identified TIV events by real-time quantitative PCR (qPCR) using RNA obtained from an independent time-course experiment (primer sequences, along with the isoforms detected by these primers, are given in File S1 sheet 2): First, qPCR using isoform-specific primers, as well as primers that amplify shared regions, was performed for ultra-high confidence (FDR<10E-15) TIV events encompassing alternative promoter usage (Figure 2C). By using isoform-specific primers, we then extended these validations in two steps, first by evaluating various types of TIV events with lower statistical stringency (Figure S2). Third, we successfully validated a random set of TIV events identified by our microarray experiment as ‘borderline’ significant with FDR values between 3% and 12% (Figure S3), which corresponds to the top 500 TIV events (compared to 373 TIV events when applying the 5% FDR threshold used in Figure 1C). For instance *HIST1H2AC* in Figure S3 is ranked 493 in the list of TIV events (File S1 sheet 1). Taken together, these experiments confirmed that TIV events could be accurately identified and validated using the employed microarray, analytic, and qPCR platforms – even when lowering the stringency of the applied FDR threshold. Moreover, we observed remarkably different – sometimes opposing – profiles of EGF-inducible transcript isoforms. Importantly, such distinct profiles occurred both for isoforms that exhibited roughly similar abundance (e.g., *FBXW7*), as well as in cases of alternative regulation of low abundance transcripts (e.g., *VAV3*; Figure 2C).

Interestingly, we observed EGF-induced alternative promoter usage of genes that have previously been implicated in tumor progression (e.g., *VEGFC*, *PTK2, IL18* and *VAV3*) or in cell survival/proliferation (e.g., *FBXW7, BID, ABL*2) [34], [35]. In addition, we detected EGF-induced switches between isoforms that have previously been associated with non-redundant functions, for example: *FBXW7* and *TSC22D1* [34], [36], [37]. Because our studies employed non-tumorigenic mammary cells, we asked whether similar alterations occur also in cancer cells. Analyses of the corresponding isoforms in a panel of breast cancer lines and in the NCI60 series of human cancer cell lines (Figures S4A and S4B) confirmed that these isoforms are expressed in the majority of cancer cell lines, and that the isoform ratios of these genes vary between cell lines. To distinguish between hard-wired isoform ratios and inducible promoter switching, we applied EGF or GW2974, a dual EGFR/ErbB-2 kinase inhibitor, on MCF10A cells, on another non-tumorigenic mammary cell line, and on nine breast cancer lines. qPCR analyses of five genes indicated rapid, kinase-dependent changes in isoform ratios in most breast cell lines mostly resembling the EGF-induced changes we observed in MCF10A cells (Figure S4C). In aggregate, these results demonstrate that the isoform ratios of these genes are cell type specific, but depend on EGFR/ErbB-2 signaling.

### EGF-induced TIV is required for mammary cell migration

**Figure 3:**
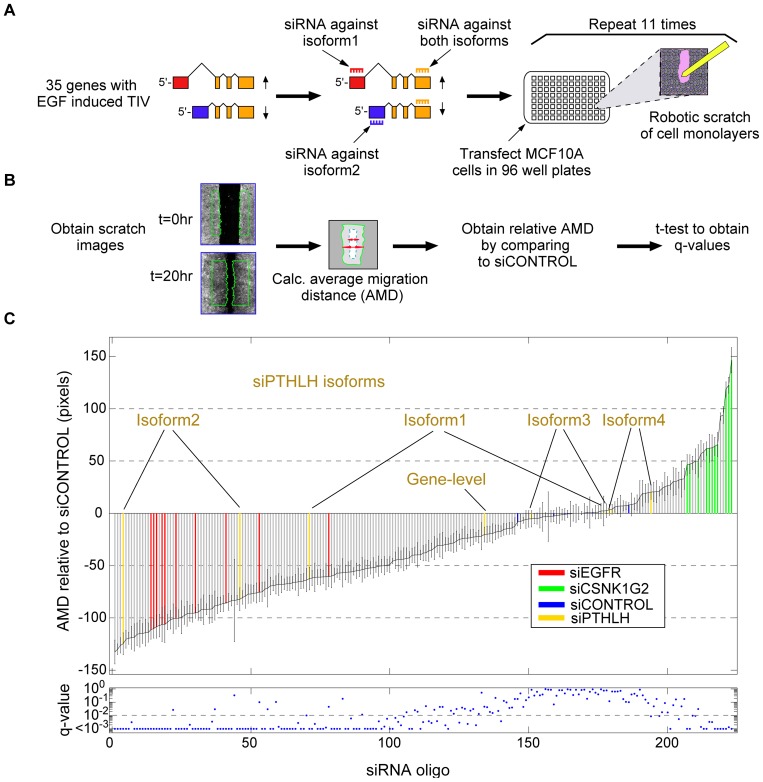
Functional tests of EGF-induced TIV by an isoform-specific siRNA screen. (**A**) For 35 genes, each transcript isoform undergoing EGF-induced TIV was targeted by two individual oligonucleotides and by ‘gene-level’ siRNA oligonucleotide pools knocking down all isoforms. An automated robotic platform was used for performing scratches. Each candidate was screened in eleven biological replicates. (**B**) Scheme of the automated image analysis used to calculate the average migration distance (AMD) for each well compared to control siRNA oligonucleotides (**C**) *Upper panel:* Each bar represents the measured mean relative AMD (and standard error) for one oligonucleotide. *Lower panel:* The FDR q-values, relative to the control siRNA, are shown.

The widespread occurrence of EGF-induced TIV suggested relevance to the motile phenotype exhibited by EGF-treated mammary cells. To test this, we performed a functional, isoform-specific RNA interference screen, using MCF10A and the scratch (‘wound closure’) assay [31], [38], [39] (Figure 3A). From the top 500 candidate genes undergoing EGF-induced TIV, candidate genes for this screen were selected based on the following criteria: TIV events *(i)* affected the protein coding sequence, *(ii)* they were detectable in at least 3 adjacent time points (‘switch-like’, rather than transient), *(iii)* the different isoforms could be specifically targeted using small interfering RNA (siRNA) oligonucleotides (oligonucleotide design is described under *Materials and Methods*), and *(iv)* availability of commercial oligonucleotide pools targeting all isoforms of the respective gene. From the resulting list of approximately 150 genes fulfilling the above criteria, we excluded those with more than 4 expressed isoforms (as detected by microarrays). From the remainder we selected 35 genes (highlighted in yellow in File S1 sheet 1), because they represent different types of TIV events, such as alternative promoter usage, cassette exon inclusion/exclusion, alternative 3′/5′ splice site choice, intron retention or alternative 3′ exons). Each expressed isoform of the selected 35 genes was specifically targeted using two distinct custom-made siRNAs (oligonucleotide sequences are listed in File S1 sheet 3), in accordance with previous siRNA screens [31], [39]. In aggregate, the number of targeted isoforms was 2, 3, and 4, respectively, in 29, 5, and 1 genes, respectively. A typical example of isoform-specific targeting of an alternative promoter usage TIV event is shown in Figure S5.

The robotized functional assay we applied included scratching of starved MCF10A monolayers, staining with a live-cell dye, stimulation with medium containing EGF and serum, and automated image capture/analysis at time zero and 20 hours after stimulation to infer the average migration distance (AMD; Figure 3B) as recently described [40]. Commercial control siRNAs, oligonucleotide pools targeting *EGFR* (impairing migration) or *CSNK1G2* (causing accelerated migration) [31], as well as commercial oligonucleotide pools targeting all isoforms of the respective genes were used as controls. For each condition eleven biological replicates were screened. The goal of this screen was to identify isoforms, whose knockdown causes phenotypic effects that are distinct from those observed with *(i)* knockdown of the alternate expressed isoforms, *(ii)* gene-level targeting of the respective gene, and *(iii)* control siRNA.

Of the 154 isoform-specific siRNAs and 35 gene-level oligonucleotide pools we screened, 95 individual isoform-specific oligonucleotides and 12 gene-level pools resulted in AMDs that significantly differed (FDR<1%) from those observed using control siRNAs (Figure 3C, File S1 sheet 4). For two genes, parathyroid hormone-like hormone (*PTHLH*) and laminin alpha 3 (*LAMA3*), oligonucleotides specifically targeting one particular isoform consistently resulted in phenotypes distinct from those observed with siRNA control, siRNAs specific to the alternate isoforms, and gene-level siRNAs. Hence, these isoform-specific phenotypes would have been missed had we applied a gene-level targeting approach. For example, only the two siRNAs targeting isoform 2 of *PTHLH* consistently inhibited migration, whereas oligonucleotides targeting the other three isoforms, or siRNAs targeting all *PTHLH* isoforms, exhibited no consistent effect (Figure 3C).

### Validation of isoform-specific knockdown and direct effects on cell migration

**Figure 4:**
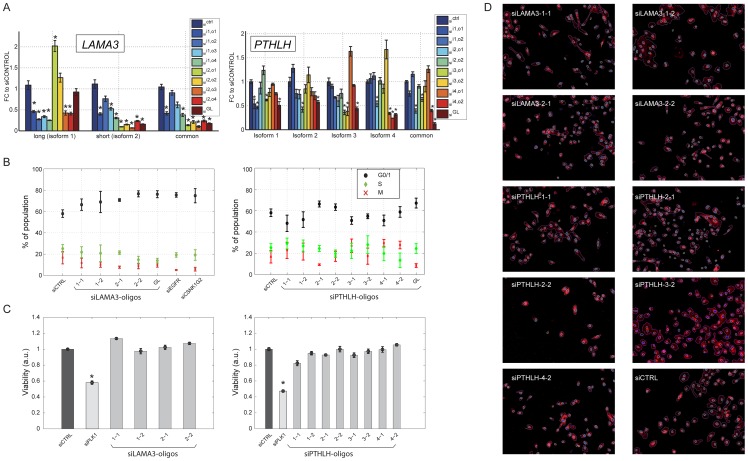
Isoform-specific knockdown and absence of significant proliferation, viability and morphological effects. (**A**) MCF10A cells were transfected with siRNA oligonucleotides (20 nM) targeting individual isoforms of *LAMA3* (left panel) or *PTHLH* (right panel), siRNA pools targeting all isoforms (‘gene-level’) or with control scrambled siRNA oligonucleotides (sictrl denotes siRNA control, ‘i1,o1’ denotes the first oligonucleotide targeting isoform 1; ‘i2,o2’ the second oligonucleotide targeting isoform 2, etc. and ‘GL’ denotes ‘gene level’). Forty-eight hours later, RNA was extracted and qPCR measurements were performed using primers specific to all expressed individual isoforms, as well as primers amplifying transcript regions common to all expressed isoforms. Measurements were normalized to results obtained with an independent siRNA control replicate. Error bars denote standard errors from three technical replicates and asterisks denote statistically significant (p<0.05) differences relative to siCtrl. Similar results were obtained in three independent repeat experiments. (**B**) Oligonucleotides for *EGFR*, which impair migration, and for *CSNK1G2*, which accelerate migration are shown as additional controls. Cell cycle analysis was performed as described in *Methods*. Error bars represent the standard errors from the analysis of 24 images taken per from each of four wells per condition. (**C**) Viability was measured by tetrazolium-based WST-1 assay. MCF10A cells were transfected with the indicated oligonucleotides (siRNA to polo-like kinase, *PLK1*, served as positive control). Error bars represent standard errors from 3 biological replicates. The experiment was performed thrice. (**D**) Shown are morphologic effects of isoform-specific oligonucleotides targeting *LAMA3* or *PTHLH*. Nine thousand MCF10A cells per well were transfected, starved and stimulated as described for the proliferation assays, followed by DAPI and phalloidin staining, 14 hours after stimulation. Automated image analysis was used to systematically assess changes in cell size and shape by analyzing 24 images taken per condition. No difference between the individual knockdowns was apparent (see also Figure S6B); one representative image for each condition is shown.

To exclude phenotypic effects due to different knockdown efficacy, we concentrated on two hits of the screen, *LAMA3*, *PTHLH*, and one other gene (*TSC22D1*; Figure S6A), and used qPCR to test effects on the targeted isoform, the alternate isoform(s), and transcript regions common to all isoforms. Beyond the oligonucleotides used in the screen, for *LAMA3* we also checked 4 additional isoform-specific oligonucleotides. As shown in Figure 4A, 17/20 (85%) and 10/20 (50%) isoform-specific oligonucleotides, respectively, knocked down their target isoform by >50% and >70%, respectively, a finding which is similar to that observed in a previous screen performed with MCF10A cells employing ‘gene-level’ targeting (in which 42% of the 321 checked oligonucleotides exhibited knockdown efficacy greater than 70%) [31]. Likewise, ‘gene-level’ oligonucleotides targeting *LAMA3* and *PTHLH* resulted in an efficient knockdown of transcript regions common to all isoforms. By contrast, despite targeting non-overlapping sequence regions (for an example, see Figure S5), the above isoform-specific oligonucleotides influenced the expression of their ‘off-target’ isoforms – an effect which could be reproduced in several independent experiments; some oligonucleotides caused up-regulation and others down-regulation of their respective ‘off-target’ isoforms, but in almost all instances the size of change was smaller than that of the targeted isoform (Figure 4A). Because sequence homology can be excluded and similar effects have recently been demonstrated by employing isoform-specific siRNAs [39], [41], we reason that this represents a compensatory mechanism, rather than a typical off-target effect. Hence, rather than selectively knocking down only one isoform, most oligonucleotides we employed skewed the ratio between the targeted isoform and the alternate isoforms in a way that led to an absolute and relative lower abundance of the targeted isoform.

Another precaution we undertook excluded indirect effects of individual *LAMA3* or *PTHLH* isoforms on cell viability or proliferation: We performed cell cycle analyses following transfection with siRNAs targeting *EGFR* (diminishing migration), *CSNK1G2* (enhancing migration), or individual *PTHLH* or *LAMA3* isoforms. In these analyses, we observed only subtle effects on proliferation (Figure 4B), which did not correlate with the effects observed in the scratch assays. To address effects on viability, we transfected MCF10A cells with isoform-specific oligonucleotides to *PTHLH* or *LAMA3*, along with control siRNAs (negative control) or oligonucleotides targeting *PLK1* (positive control), and performed cell viability (WST-1) assays. In these tetrazolium-based assays, only minimal effects of the siRNAs on viability were observed (Figure 4C). Finally, to assess yet another potential confounding effect on cell morphology, we transfected MCF10A cells with control siRNA oligonucleotides or siRNAs targeting *EGFR*, *CSNK1G2* or individual *PTHLH* or *LAMA3* isoforms. Actin and nuclei were then stained with phalloidin and DAPI, respectively, followed by automated image capture and analyses that measure cell size and shape. These analyses revealed no large or consistent morphological effects of the siRNA oligonucleotides (Figure 4D and Figure S6B), in line with functional specificity of our migration screen.

### EGF-induced transcript isoforms exhibit context-specific roles

**Figure 5:**
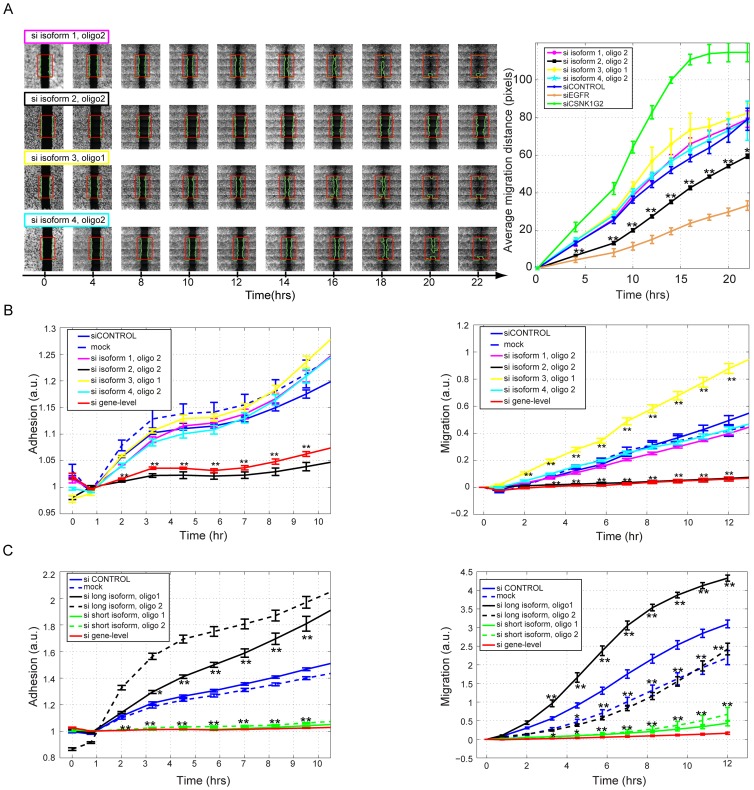
Validation of hits of the siRNA screen. (**A**) Time lapse microscopy images (one of 5 repeats) of scratch assays using MCF10A cells transfected with isoform-specific siRNAs to *PTHLH* (left) and quantification of all images (right). Error bars represent the standard deviations of five repeats per condition; asterisks indicate significant differences from siCONTROL (*p<0.05, **p<0.01). (**B** and **C**) Results of Real-time Cell Analyzer (RTCA) experiments measuring the effects of isoform-specific oligonucleotides to *PTHLH* (B) or to *LAMA3* (C) on cell adhesion (left panels) and single cell migration through filters (right panels). Oligonucleotides targeting transcript regions common to all expressed isoforms of the respective gene were also employed (si gene-level). Cells treated with transfection reagent alone (‘mock’) and cells transfected with scrambled control siRNA oligonucleotides (siCONTROL) are shown as controls. Error bars represent the standard deviations of 3 or more repeats per condition; asterisks indicate significant differences relative to siCONTROL (*p<0.05, **p<0.01). The experiment was repeated thrice.

To better comprehend the migration-specific effects of EGF-induced TIV events that emerged from our screen, we performed time-lapse imaging of scratch assays and determined AMD as a function of time. This survey revealed that individual isoforms of *PTHLH* and *LAMA3* exhibited distinct effects on migration speed, as exemplified for *PTHLH* isoforms in Figure 5A. By contrast, migration onset, cell-cell contacts, as well as the collective mode of epithelial migration, were not appreciably affected. Interestingly, however, siRNAs targeting the short *LAMA3* isoforms – isoform 2– not only impaired MCF10A migration, but recurrently caused detachment of the epithelial rim close to the scratch site suggesting an role of this isoform in MCF10A cell adhesion to the surface.

Therefore, the effects of *PTHLH* and *LAMA3* isoforms on cell adhesion and individual cell migration were further investigated by using a Real-Time Cell Analyzer (RTCA). For adhesion assays, the RTCA device measures time-resolved changes in the electrical impedance induced by cell adhesion to a charged surface, whereas.for single-cell migration assays the RTCA measures impedance changes induced by cells that migrate through a porous membrane and subsequently adhere to the charged lower surface of the membrane (see *Materials and Methods*). Evaluation of the functional roles of individual *PTHLH* isoforms by these assays revealed that like in collective cell migration (scratch) assays, knockdown of isoform 2 strongly inhibited both adhesion (Figure 5B; left panel) and individual cell migration of MCF10A cells (Figure 5B; right panel). Interestingly, in these single-cell migration assays, knockdown of isoform 3 promoted migration, an effect that was not apparent in the collective cell migration assay employed in our siRNA screen. RTCA experiments performed after knockdown of individual *LAMA3* isoforms confirmed our notion from scratch assays that targeting the short *LAMA3* isoforms (isoform 2), which are up-regulated by EGF, significantly impaired adhesion of MCF10A cells. By contrast, knockdown of the long, EGF-downregulated isoforms (isoform 1), promoted adhesion of suspended MCF10A cells (Figure 5C, left panel). Evaluation of additional pairs of siRNAs specific for each isoform (shown as i1_o3, i1_o4, and i2_o3, i2_o4, respectively, in Figure 4A) confirmed that targeting the short isoforms impairs adhesion (Figure S7). Notably, one of the additional oligonucleotides targeting the long isoforms was also found to inhibit MCF10A adhesion, whereas the other one exhibited no significant phenotypic effect (Figure S7). Likewise, in single-cell migration assays all four oligonucleotides targeting short *LAMA3* transcripts (like gene-level pools) diminished migration. Individual oligonucleotides targeting the long *LAMA3* isoforms, however, again revealed somewhat discordant results, with three oligonucleotides targeting these isoforms resembling the effect of control siRNA, and one inhibiting MCF10A migration (Figure 5C; right panel, and Figure S7).

In aggregate, the combination of experiments employing a scratch-assay and time-lapse microscopy, which follow collective cell migration, and the complementary adhesion and transwell assays, which track individual modes of cell migration, confirm the functional non-redundancy of *LAMA3* and *PTHLH* isoforms. Likewise, the phenotypic effects of individual transcript isoforms that became apparent in different migratory contexts, underscore the critical roles played by TIV in inducible locomotion.

## Discussion

Transcriptional responses to extracellular signals are commonly described in terms of the number and identity of up- or down-regulated mRNAs [42]. Herein, we report that in response to a stimulus, cells employ a rapid, often non-monotonous, shift of their transcript isoform composition. The occurrence of different types of TIV in response to a defined cue, along with their distinct temporal dynamics, suggests that these events are governed by several, mechanistically distinct, regulatory processes. Beyond the remarkably large number of isoforms we observed and their unexpectedly wide variation, the results of our isoform-specific siRNA screen demonstrate that stimulus-induced TIV plays non-redundant, crucial roles in establishing the migratory phenotype of growth factor-stimulated mammary cells. Interestingly, our screen detected a surprisingly large number of siRNA oligonucleotides able to impair migration. We attribute this large number of hits – in comparison to similar screens that used gene-level targeting without prior assessment of the expression of target genes [31], [38] – to the fact that all our targets were stringently pre-selected; not only were they unambiguously expressed, but they were also strongly regulated by the pro-migratory EGF stimulus at the mRNA level. Arguably, in our study the probability of phenotypic hits is higher than for a random set of screened genes. Moreover, as shown in Figure 3A, our screen consisted of eleven repeats. Hence, for some isoforms even relatively small phenotypic effects could be identified with high statistical significance.

Another interesting aspect of our study encompasses the knockdown effects of isoform-specific oligonucleotides. The majority of isoform-specific siRNAs efficiently down-regulated their respective target isoforms. To a lesser extent, however, and likely mediated by compensatory changes, these oligonuleotides also reproducibly up- or down-regulated individual ‘off target’ isoforms of the same gene, in agreement with previous observations [41]. Irrespective of the exact mechanism responsible for these changes, our approach demonstrates that preferential targeting of specific isoforms was achieved and that the relative abundance of different transcript isoforms bears essential functional importance.

The strongest effects of individual transcripts on cell migration were found when testing isoforms of *LAMA3* and *PTHLH*. LAMA3 is a secreted protein required for formation of basement membranes and hemidesmosomes, as well as for the establishment of focal contacts, instigation of cell migration and promotion of wound healing (reviewed in [43]). Accordingly, *LAMA3* expression is controlled by specific growth factors [43], and it is mediated by two promoters, regulating long and short isoforms [44]. Moreover, defects in cell adhesion and in collective cell migration might relate to yet a third isoform, which results from transcription of the same promoter as the long isoform described herein, but exhibiting premature transcript termination, suggesting that this isoform plays a dominant negative role [45]. While our analyses detected no alterations of the latter, we found that the long *LAMA3* isoforms are downregulated by EGF and they inhibit mammary cell adhesion and migration. By contrast, we found that the short, EGF-induced *LAMA3* transcripts are required for MCF10A cell adhesion and migration. Consistent with a positive role in tumor progression, expression of the short *LAMA3* isoforms, which is multiply controlled by the transcriptional co-activator EP300 [46], along with the transcription factors AP-1 [47], CREB1 [48] and USF1 [49], is increased and portends a dismal prognosis in head and neck cancers [50]. In conclusion, our data indicates that a single gene, *LAMA3*, encodes transcripts able to either promote or inhibit cell migration, in line with the assertion that analyses of inducible transcription would need to resolve the kinetics of individual transcript isoforms.

Like LAMA3, parathyroid hormone-like hormone (PTHLH, also PTHrP) is a secreted factor. PTHLH is critically involved in smooth muscle contraction, as well as in development of several organs, including enchondral bone, skin, teeth and the mammary gland. Tumor-associated secretion of PTHLH is causally implicated in malignancy-associated hypercalcemia, as well as in the initiation, progression and metastasis of breast cancer [51]. At least four well-annotated *PTHLH* transcript isoforms are generated by alternative usage of two promoters and by alternative mRNA splicing [52]. Cancer-specific differential expression *PTHLH* transcript isoforms has been reported in prostate and in other cancers [53], and the ratio of *PTHLH* isoforms has been associated with breast cancer outcome [54]. Moreover, EGF signaling has been reported to alter the relative ratios of these isoforms, but the molecular mechanisms underlying these changes, along with the functions of specific transcripts, remained unknown [55]. Employing EGF-stimulated mammary cells, we found that all four isoforms are expressed in MCF10A cells. Moreover, the results of our siRNA survey assigned a specific function in collective cell migration to isoform 2 of *PTHLH* (Figures 3C and 5A). Since PTHLH represents a potential therapeutic target in breast and in other types of cancer [51], we assume that further studies will reveal additional, isoform-specific roles susceptible for pharmacological intervention.

In aggregate, the results we obtained using normal human mammary cells and several cancer cell lines suggest that rather than representing a stochastic transcriptional phenomenon, stimulus-induced TIV represents a rapid, orchestrated transcriptional response that critically determines the phenotypic response to extracellular cues, like EGF.

## Materials and Methods

### Reagents and cell lines

Unless indicated, reagents were from Sigma-Aldrich (Rehovot, Israel). Cell lines (ATCC, Manassas, Virginia) were cultured as recommended by the supplier. For microarray and qPCR experiments, MCF10A cells were growth-factor and serum-starved and stimulated with EGF-containing (20 ng/ml) starvation medium as described previously [33]. Likewise, total RNA isolation, as well as sample processing for microarrays and real-time quantitative PCR (qPCR) were performed as described [33].

### Microarray data analysis

We used Affymetrix GeneChip® Human Exon 1.0 ST (Santa Clara, CA) microarrays, which interrogate the expression of both constitutive and putative exonic transcript regions. The 1.4 million probe sets (PS) of each array are divided into three types: ‘core’ (∼280 K PS; supported by Ref Seq transcripts and full-length mRNAs), ‘extended’ (additional ∼520 K PS; supported by cDNA-based annotations) and ‘full’ (additional ∼580 K PS; supported by *ab initio* gene predictions). Our analysis considered the ‘full’ set and used minimal assumptions about the underlying transcript isoform models. This was accomplished by several steps. First, we estimated the signal distribution arising from intronic transcript regions. This was used to define truly exonic transcript regions, thereby reduced false positive TIV predictions and enabling discovery of new TIV. Second, we used three biological replicates and intensity-dependent noise estimation [56], along with filtering of PS with suboptimal properties and adjusting for multiple testing. These resulted in a more accurate estimation of expression noise compared to studies using a standard estimator of the expression variance within groups or between replicates. The detailed steps of microarray data processing and analysis are described under Information S1. Microarray data have been deposited under Gene Expression Omnibus (GEO; GSE24391). For analyses of the NCI60 panel and of breast cancer cell lines, we used publically available microarray datasets from GEO (GSE29682) and Array Express (E-MTAB-181).

### RNA interference

Candidate genes were selected as described under *Results*. For screening purposes, two oligonucleotides per isoform were chosen as a tradeoff between the number of required oligonucleotides, the risks of false-negatives emanating from ineffective oligonucleotides, and of false-positives due to off-target effects [31], [39]. Upon receipt of FASTA target transcript sequences, Qiagen (Frederick, Maryland, USA) custom designed 8 potential siRNA oligonucleotide sequences for each isoform [57]. From these, we selected two oligonucleotides per isoform, which satisfied the following criteria: Predicted efficacy score >0.75 (based on BIOPREDsi [58]), absence of low sequence complexity, SNP sites and immunostimulatory motifs, respectively, absence of 4-mer repeats, and less than 7 base-pair sequence complementarity to the off-target isoforms (except for the exclusion isoforms of cassette exon inclusion/exclusion events, where up to 15 nucleotides sequence complementarity was allowed). As controls, the commercial non-targeting siAllStars, the migration-activating control si*CSNK1G2*, and the migration-inhibiting control si*EGFR* oligonucleotide pools were used, along with commercially available gene-level siRNA pools targeting all isoforms of the selected 35 genes (Qiagen, Hilden, Germany).

### Transfections and scratch assays

For wound-healing scratch assays, 5.5×10^4^ MCF10A cells per well were plated without antibiotics in black-walled 96-well glass-bottom plates (GE MatriCal, Spokane, Washington). The cells were transfected sixteen hours later with siRNAs (final concentration 40 nM) and Lipofectamine 2000 transfection reagent (0.75 µl/well; Invitrogen, CA, USA) diluted in Optimem (Invitrogen). Twenty-four hours later, the medium was switched to starvation medium [33]. After another 24 hours, MCF10A monolayers were stained with CellTracker Green CMFDA live-cell dye (Life Technologies, Darmstadt, Germany) and wounded by generating a longitudinal scratch using the 96-well pipetting device of a BiomekFX pipetting robot (Beckman Coulter, Krefeld, Germany). Thereafter, cells were washed once with EGF- and serum-containing medium and plates were scanned with the 10X objective of an Olympus ScanR microscope (Olympus SIS, Munster, Germany) in the YFP-channel, to determine the initial scratch width, as well as the scratch width after 20 hours. For each well, 30 sub-images were acquired. For time-resolved analysis of wound healing, cells were treated as described above, but kept in the incubator associated with the microscope (resulting in somewhat faster migration) and scanned at the time points indicated in Figure 5A.

### Assessment of cell viability, proliferation and morphology

For assessment of viability effects, cells were transfected with the indicated oligonucleotides and starved as described for scratch assays, followed by stimulation with EGF- and serum-containing medium for 8 hours. Metabolic activity was then measured by the tetrazolium-based WST-1 Cell Proliferation Reagent (Roche Applied Science, Mannheim, Germany) using siRNA to *PLK1* as positive control [59]. For measurements of cell proliferation, 12×10^4^ cells/well were plated in 96 well glass bottom plates. Twenty-four hours later, cells were transfected with either control siRNA oligonucleotides (‘allstar’), siRNA against *EGFR* (which inhibits MCF10A migration), siRNA against *CSNK1G2* (which enhances MCF10A migration) or siRNAs targeting individual or all isoforms of *LAMA3* and *PTHLH*, respectively. Cells were then incubated overnight in serum and EGF-containing medium, followed by 24 hours of incubation in starvation medium. Thereafter, cells were stimulated with serum-containing medium supplemented with EGF (20 ng/ml) for 12 hours, followed by DAPI staining (1 µg/ml) of adherent cells. Twenty-four images were taken per well (10× magnification) as described for the scratch assay, and the ScanR Olympus analysis software was used for analysis.

### Image analysis

We developed a dedicated software package for image analysis of scratch assays used in the siRNA screen and time-lapse microscopy of screen hits [40]. In brief, for each well and each time point 24 (6×4) high magnification fluorescent microscopy images were acquired. Sub-images were merged into a single image that was converted to gray scale, followed by segmentation. The upper and lower 15% of the image were discarded (see green frame in Figure 3) to analyze only the homogeneous central portion of scratches, and a frame was set at the image center. The contour of migrating cells was identified, followed by average gap width measurement for each time point. The average migration distance (AMD) was quantified by relating the gap width measured at the respective time points to the initial width (t = 0).

### Adhesion and transwell migration assays

Cell attachment and transwell migration assays were performed using an xCELLigence Real Time Cell Analyzer (RTCA) DP system (Roche, Penzberg, Germany). The device measures time-resolved electrical impedance changes resulting from cell attachment to electrodes located on the plate surface (E-plate; adhesion assay). Alternately, when Boyden chambers with electrodes located at the lower side of a porous membrane (denoted CIM-plates) are used, transwell cell migration can be assessed in real-time. All measurements were quantified by the arbitrary unit Cell Index and they reflect the number of attaching cells and the relative strength of their attachment. For all RTCA assays, MCF10A cells were seeded in 6-well plates, transfected with 40 nM (final concentration) of the indicated siRNA oligonucleotides using 4 µl Lipofectamine 2000 per well in 1 ml final volume. Forty-eight hours after transfection, starved cells were carefully trypsinized and counted using a *CASY* cell counter (CASY-DT-2CB, Roche Innovatis, Bielefeld, Germany). For analysis of cell attachment, 4 replicates of 1.0×10^4^ cells per well (100 µl) were seeded in EGF-containing propagation medium in a 16-well E-plate. The first step consisted of background determination, followed by impedance measurement for 12 h, in 15 min intervals, to measure cellular attachment. For analysis of transwell cell migration, 1.0×10^5^ cells per well were seeded in propagation medium without EGF (100 µl), into the upper compartment of a 16-well CIM-plate in 6 replicates. The lower compartment was filled with EGF-containing propagation medium (175 µl), and the chambers were assembled. The next step enabled background reading, followed by impedance measurement for 24 h (in 15-min intervals) to measure attachment of cells, which migrated through the membrane (8 µm pore size) to the other side (bottom).

## Supporting Information

Figure S1 **Identification of true exonic PS and TIV events.** (**A**) Signal (log2 scale) distribution of *‘absent’* and *‘present’* PS according to the Affymetrix detection p-value. (**B**) Noise (standard deviation) versus signal intensity, as estimated from the biological triplicates of each time point. (**C**) Signal distribution of potential exons and constitutive introns (according to the UCSC gene model) of those PS that passed all the filtering steps. (**D**) Distribution of the difference between the average exon signal and the average intron signal (within the same gene) before and after applying the corrected exon/intron definition; note that before and after the correction 83% and 98%, respectively, of the values are greater than zero.
(EPS)

Figure S2 **qPCR validation of additional EGF-induced TIV events.** The following EGF-stimulated events (MCF10A cells) are exemplified: alternative promoter usage (*FRMD4B*), cassette exon exclusion/inclusion (*ASAP2*), intron retention (*TMEM138*) and both alternative promoter usage and alternative 3′ transcript end formation (*PTHLH*). Note the unique dynamics of *FRMD4B* (i.e., different isoforms peaking at different times). Error bars represent standard deviations from three technical replicates.
(EPS)

Figure S3 **qPCR validation of ‘low-confidence’ EGF-induced TIV events detected by microarrays.** qPCR experiments were performed as in Figure S3, but for genes exhibiting ‘borderline confidence’ (i.e., FDR 3–12%) TIV events according to FDR-based microarray analyses (for which a cut-off of 5% was used). Events represent alternative 3′ splice site choice and intron retention (*ABCC5*), alternative promoter usage (*HIST1H2AC*, *TINAGL1*, *TMCC1*, *ZNF451*), alternative 3′ transcript end formation (*POLR1C*).
(EPS)

Figure S4 **Expression and dynamic behavior of EGF-induced isoforms encoded by alternative promoters in cancer cell lines.** (**A** and **B**) Heat maps displaying the ratios of the short to long isoforms for the genes (rows) shown in Fig. 2B across a panel of breast cancer cell lines (A), and across the NCI60 panel of cancer cell lines (B). Shown are all genes in which both isoforms were clearly detectable in all cell lines; the remaining genes are in white. Note that most isoforms of the same gene that switched their relative abundance in MCF10A cells upon EGF stimulation are also expressed at different ratios in most cell lines. (**C**) The indicated mammary cell lines (columns) were treated with EGF (20 ng/ml), the dual specificity EGFR/ErbB2 kinase inhibitor GW2974 (1 μM), or solvent (untreated; ‘UT’) for 4 hours as indicated by the bar right to the heat map. Next, qPCR was used to interrogate the relative expression of isoforms of five genes, whose isoforms are encoded by alternative promoters and displayed alterations upon EGF stimulation of MCF10A cells (see Fig. 2B). The heat map displays the fold change ratio (log2 scale; normalized separately for every gene and cell line) of the isoforms. Note that in most cell lines, the isoform ratios of the genes presented exhibited variability upon application of EGF and GW2974, and the direction of these changes was mostly concordant with that observed in MCF10A cells.
(EPS)

Figure S5 **Positions of** ***LAMA3*** **isoform-specific siRNA oligonucleotides and primers.** *Upper panel:* Organization of LAMA3 transcript isoforms, shown as in Figure 2A. Transcript positions corresponding to siRNA oligonucleotides specific to the long (siLAMA3–1–1 and siLAMA3–1–2) or to the short *LAMA3* isoforms (siLAMA3–2–1 and siLAMA3–2–2) are shown in red (the uppermost isoform was not differentially expressed in MCF10A cells upon EGF stimulus). *Lower panel:* The magnified regions indicate the positions of primers corresponding to specific isoforms (qPCR primers *LAMA3* long isoforms, qPCR primers *LAMA3* short isoforms) or transcript regions common to all isoforms (qPCR primers *LAMA3* common).
(EPS)

Figure S6 **Efficacy and specificity of isoform-specific siRNA oligonucleotides.** (**A**) MCF10A cells were transfected separately with two siRNA oligonucleotides (40 nM) targeting individual isoforms of *TSC22D1* or with control scrambled siRNA oligonucleotides (sictrl denotes siRNA control, ‘i1,o1’ denotes the first oligonucleotide targeting isoform1; ‘i2,o2’ the second oligonucleotide targeting isoform 2, etc.). Forty-eight hours later, RNA was extracted and qPCR measurements were performed using primers specific to all expressed individual isoforms, as well as primers amplifying transcript regions common to all expressed isoforms. Measurements were normalized to results obtained with siRNA control. Error bars denote results from three technical replicates, and asterisks denote p-values <5%. Similar results were obtained in two independent repeat experiments. (**B**) Cell size distribution of MCF10A cells transfected treated as described in legend to Figure 4D, calculated by automated image analysis. Controls included siRNA oligonucleotides to *EGFR* (which inhibit MCF10A migration), to *CSNK1G2* (which accelerated MCF10A migration) and scrambled siRNA oligonucleotides (siCTRL; no phenotypic effect).
(EPS)

Figure S7 **Relevance of individual** ***LAMA3*** **transcript isoforms for MCF10A cell adhesion and single-cell migration.** Graphs represent Real-time Cell Analyzer (RTCA) results of adhesion (left) and transwell migration (right) assays of MCF10A cells transfected with additional siRNA oligonucleotides (distinct from those employed in Figure 5C) specifically targeting the indicated *LAMA3* isoforms. Oligonucleotides targeting transcript regions common to all expressed *LAMA3* isoforms were also employed (si gene-level). Cells treated with transfection reagent alone (‘mock’) and cells transfected with scrambled control siRNA oligonucleotides (siCONTROL) are shown as controls. The experiment was repeated twice.
(EPS)

Information S1 Supporting Methods; Supporting Results; Supporting References.
(DOCX)

File S1 Sheet 1: microarray results. Sheet 2: primer sequences. Sheet 3: siRNA oligonucleotides sequences. Sheet 4: siRNA screen results.
(ZIP)
