# Supplementary material for: Epidermal Growth-Factor – Induced Transcript Isoform Variation Drives Mammary Cell Migration
Source: PLoS One. 2013 Dec 6;8(12):e80566. doi: 10.1371/journal.pone.0080566 (PMC3855657; doi:10.1371/journal.pone.0080566)
Supplement: Information S1 — Supporting Methods; Supporting Results; Supporting References. (DOCX) [file pone.0080566.s008.docx]

**Supporting Information for: Epidermal growth-factor - induced transcript isoform variation drives mammary cell migration**

*Wolfgang J. Köstler, Amit Zeisel, Cindy Körner, Jonathan M. Tsai, Jasmine Jacob-Hirsch, Nir Ben-Chetrit, Kirti Sharma, Hadas Cohen-Dvashi, Assif Yitzhaky, Eric Lader, Ulrich Tschulena, Gideon Rechavi, Eytan Domany, Stefan Wiemann and Yosef Yarden*

**Supporting Methods**

**Microarray data processing, the theory and practice of TIV event identification**

Pre-filtering included only the 669,554 PS that perfectly match a unique genomic sequence that is both known to be transcribed (’crosshyb type=1’), and has a gene symbol (UCSC genes, http://genome.ucsc.edu, NCBI36/Hg18 assembly). For filtering, we first used the PS p-value provided by the Affymetrix software (similar to the MAS5 algorithm). Thereafter, the FDR procedure (at level of 5%) was used for each array to flag individual PS as *’absent’* or *’present’*. The distribution of signals according to this p-value is presented in Figure S1A. Next, a ’*noisy flag*’ was attached to PS whose standard deviation (SD) within the three biological replicates was significantly higher (passed a test of 10% FDR) than the SD expected from the noise estimation [[1](#_ENREF_1)]. Finally, a threshold of 5 (log scale) was used as the minimal intensity with a reasonable signal to noise ratio. A PS passed the filtering at a specific time point if: (i) at least two out of the three repeats were marked as ’present’, (ii) the PS was not flagged as noisy, (iii) the mean intensity over the ’present’ signals was above the signal threshold. Next, noise was estimated in an intensity-dependent manner [[1](#_ENREF_1)] from the triplicates measured at each time point (Figure S1B).

The challenge of correctly identifying TIV events in total RNA samples obtained in time course experiments, namely under transcriptional non-steady state conditions, lies in the correct discrimination between those putative exons that represent TIV events and PS interrogating transcript regions that are intronic under all conditions. Because the latter interrogate pre-mRNA, their dynamic behavior is often dramatically different from that of constitutive exons in the course of a transcriptional response to a stimulus [[2](#_ENREF_2)]. In order to use a minimal number of assumptions about the definition of a transcribed region as being exonic or intronic, whilst reducing the chance of discovering false positive TIV events, the UCSC gene model was used to learn about expression levels of PS interrogating transcript regions defined as constitutively ’intronic’ (i.e., which belong to intronic regions in all isoforms included in the respective gene model). We assumed that the majority of the latter would indeed be introns. In fact, PS from intronic regions have low expression values relative to those from exonic regions (Figure S1C). Hence, we used the constitutive introns as representative of intron behavior, and retained only those potential exons that exhibited significantly higher expression levels than the introns of the same gene, in at least two adjacent time points (Figure S1D). Genes represented by >4 exonic PS meeting the above definitions were considered expressed. Thereafter, the gene level FC (compared to unstimulated conditions) was estimated as the median FC of those exonic probes along each gene.

The null hypothesis for identification of TIV events is that all changes of expression are of the same magnitude for all isoforms (i.e., the relative abundance of isoforms did not change). Owing to technical noise and biological variability, even under the null hypothesis, the FC of exonic PS of the same gene displayed some distribution. We defined a PS p-value for each exonic PS, which reflects the probability of obtaining the observed inconsistency by chance, assuming that the null hypothesis is true. As a first step, we estimated the distribution and the means FC of the PS of the same gene under the null hypothesis. Noting that the variance of the PS FC for the same gene depends on the PS expression level, we used our previously developed procedure [[1](#_ENREF_1)] to estimate the width of the PS FC distribution, and tested for the probability of getting, under the null hypothesis, the observed FC value or higher, producing for every PS a p-value for being part of a TIV event.

We denote:

*A_jik_* = as the log expression of PS *j* in gene *i* at time point *k* = 1**,** 2,,,, 7 then *F_ji_ = A_ji1_ − A_ji2_* is the log FC of PS *j* in gene *i* between time points 1 and 2. According to the null hypothesis and the above assumptions:

Accordingly, the PS p-value is obtained from the normal cumulative distribution function (CDF). In such a formulation the resulting p-values are not completely independent because each PS affects the median, but this dependency becomes weak when the number of PS is large.

Next, we calculated the gene level TIV p-value, which represents the significance of the set of PS p-values from the same gene. At this stage we analyzed only genes with more than four expressed exonic PS that passed the filtering at the two compared time points. For each time point *t_i_*, pairwise comparison with *t*=0 yielded a set of *n* PS p-values (*p_1,t_, p_2,t_, . . . p_n,t_*) . We used the Fisher inverse chi-square meta p-value (reviewed in [[3](#_ENREF_3)]) to produce the gene level TIV p-value. We noticed that when producing the gene level p-value, an efficient way to overcome the noisy nature of the expression measurement was to use the mean PS p-value observed at two adjacent time points. This heuristic reduces the number of false positives and prevents identification of transient TIV effects as significant. For each gene, we calculate the gene level statistic after obtaining the PS p-values for two adjacent time points ({*p_1,t1_, p_2,t1_ , . . . p_n,t1_*}, {*p_1,t2_, p_2,t2_ , . . . p_n,t2_*}) :

**
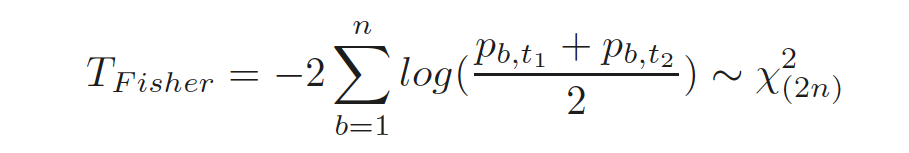
**

The gene level TIV p-value is obtained by the chi-square cumulative distribution function. Finally, we calculated the FDR for the gene level TIV p-values obtained at all time points to declare the significant candidate genes. By measuring gene level TIV p-values for each gene along the time course, we identified the peak time point of the TIV event.

**Supporting Results**

**Functional enrichment analyses**

Functional enrichment analyses were performed using the DAVID functional annotation tool (<http://david.abcc.ncifcrf.gov/>). We pasted all genes we identified as undergoing TIV upon EGF stimulation (with an FDR threshold <10%, default options and as background we used: species-homo sapiens, Affymetrix HuEx-1_0-st-v2 arrays) listed in File S1 sheet 1, and looked for enrichment in the following annotation categories: GeneOntology cellular components (GOTERM_CC_FAT), molecular functions (GOTERM_MF_FAT) or biological processes (GOTERM_BP_FAT), along with PANTHER molecular functions, biological processes, and pathways, KEGG pathways, INTERPRO and SMART protein domains (for all analyses applying an FDR threshold of 10% to adjust for muliple testing), respectively. In these analyses, only the following rather general GO terms were significantly enriched in our gene list (with 446/456 genes having a DAVID gene ID): ‘extrinsic to membrane’ (GOTERM_CC_FAT), ‘cytoskeletal protein binding’ ‘purine ribonucleotide binding’, ‘ribonucleotide binding’ (GOTERM_MF_FAT). Hence, in the list of genes undergoing TIV in response to EGF there was no clear enrichment in genes/processes commonly associated with proliferation, survival or migration.

**Supporting References**

1. Zeisel A, Amir A, Kostler WJ, Domany E (2010) Intensity dependent estimation of noise in microarrays improves detection of differentially expressed genes. BMC Bioinformatics 11: 400.

2. Zeisel A, Kostler WJ, Molotski N, Tsai JM, Krauthgamer R, et al. (2011) Coupled pre-mRNA and mRNA dynamics unveil operational strategies underlying transcriptional responses to stimuli. Molecular Systems Biology 7: 529.

3. Hong F, Breitling R (2008) A comparison of meta-analysis methods for detecting differentially expressed genes in microarray experiments. Bioinformatics 24: 374-382.
